# The Immunological Contribution of a Novel Metabolism-Related Signature to the Prognosis and Anti-Tumor Immunity in Cervical Cancer

**DOI:** 10.3390/cancers14102399

**Published:** 2022-05-13

**Authors:** Sihui Yu, Xi Li, Mingjun Ma, Rui Yang, Jiawen Zhang, Sufang Wu

**Affiliations:** Department of Obstetrics and Gynecology, Shanghai General Hospital, School of Medicine, Shanghai Jiao Tong University, Shanghai 201620, China; sihui_2009@sjtu.edu.cn (S.Y.); lx_684594@sjtu.edu.cn (X.L.); sj-mmj@sjtu.edu.cn (M.M.); yangrui119@sjtu.edu.cn (R.Y.); Reproductive Medicine Center, Department of Obstetrics and Gynecology, Shanghai General Hospital, School of Medicine, Shanghai Jiao Tong University, Shanghai 201620, China

**Keywords:** cervical cancer, metabolism-related genes, risk score signature, prognostic model, immune infiltration

## Abstract

**Simple Summary:**

Cervical cancer is the most commonly diagnosed gynecological malignant carcinoma worldwide. It is crucial to develop reliable prognostic models to predict clinical outcomes and identify patients who will benefit from different treatment strategies. In current study, we identified a reliable metabolism-related signature composed of ALOX12B, CA9, FAR2, F5 and TDO2 for the prognosis and anti-tumor immunity in cervical cancer. Patients with high-risk score underwent apparently worse prognosis and displayed lower infiltration of tumor infiltrating lymphocytes. Additionally, the metabolism-based risk score could also predict the prognosis of patients with cervical cancer based on the expression of immune checkpoints. Since this risk score signature achieves a good performance in predicting clinical outcome, we genuinely expect that our study could provide an effective prognostic tool for guidance of personalized treatment of cervical cancer patients.

**Abstract:**

Cervical cancer is the most frequently diagnosed malignancy in the female reproductive system. Conventional stratification of patients based on clinicopathological characters has gradually been outpaced by a molecular profiling strategy. Our study aimed to identify a reliable metabolism-related predictive signature for the prognosis and anti-tumor immunity in cervical cancer. In this study, we extracted five metabolism-related hub genes, including ALOX12B, CA9, FAR2, F5 and TDO2, for the establishment of the risk score model. The Kaplan-Meier curve suggested that patients with a high-risk score apparently had a worse prognosis in the cervical cancer training cohort (TCGA, *n* = 304, *p* < 0.0001), validation cohort (GSE44001, *n* = 300, *p* = 0.0059) and pan-cancer cohorts (including nine TCGA tumors). Using a gene set enrichment analysis (GSEA), we observed that the model was correlated with various immune-regulation-related pathways. Furthermore, pan-cancer cohorts and immunohistochemical analysis showed that the infiltration of tumor infiltrating lymphocytes (TILs) was lower in the high-score group. Additionally, the model could also predict the prognosis of patients with cervical cancer based on the expression of immune checkpoints (ICPs) in both the discovery and validation cohorts. Our study established and validated a metabolism-related prognostic model, which might improve the accuracy of predicting the clinical outcome of patients with cervical cancer and provide guidance for personalized treatment.

## 1. Background

Cervical cancer is the most commonly diagnosed gynecological malignant carcinoma and accounts for an estimated 604,000 new cases and 342,000 deaths annually worldwide [1]. During the past few decades, although cervical cancer screening programs and comprehensive treatment strategies, including emerging anti-tumor immunotherapy, have reduced the incidence and mortality rates in most areas of the world, the prognosis of advanced cervical cancer patients is still not improved. Therefore, it is crucial to develop reliable prognostic models to predict clinical outcomes and identify patients who will benefit from different treatment strategies.

Metabolic reprogramming in cells and changes in energy metabolism levels have been identified as an emerging hallmark of cancer [2,3]. Increased aerobic glycolysis, fatty acid (FA) metabolism and glutamine decomposition contribute to malignant transformation, the invasion-metastasis cascade, tumor microenvironment (TME) stress and the treatment resistance of cancers [4,5,6,7]. Previous studies have shown that the Warburg effect and mitochondrial dysfunction favored the metabolic adaptation and survival of cervical cancer cells [8], and that reprogramming of fatty acid metabolism was associated with lymph node metastasis of cervical cancer [9]. Meanwhile, metabolic profiles could also distinguish cervical precancerous lesions from the normal cervical epithelium [10].

It is increasingly clear that crosstalk between abnormal metabolism and immune escape assumes a key role in the process of tumor progression [11,12]. For instance, cervical cancer cells can secrete lactate to convert the phenotype of tumor macrophages [13]. Recently, the application of bioinformatic analysis in predicting the prognosis and treatment response of patients with a malignant tumor has attracted rising attention. Within the context, some researchers have combined metabolomics with genomics to demonstrate the relationship between metabolism and immune infiltration [14,15,16]. However, to date, the association between the metabolism-based risk score model and TME landscape in cervical cancer remains uncharted territory.

In the current study, we established a novel prognostic metabolism-related risk score based on cancer genomics, bioinformatics and immunohistochemical analysis. The association between the risk score and infiltration of tumor infiltrating lymphocytes (TILs) was explored in a cervical cancer training cohort, a validation cohort and pan-cancer cohorts. Moreover, we also utilized the risk score to predict the prognosis of cervical cancer patients in the context of different expression of immune checkpoints.

## 2. Materials and Methods

### 2.1. Data Retrieval and Identification of Differentially Expressed Metabolism-Related Genes

The metabolism-related gene sets were downloaded from the Molecular Signature Database (MSigDB) via the Gene Set Enrichment Analysis tool (GSEA, http://software.broadinstitute.org/gsea/index.jsp (accessed on 3 May 2021)). The RNA sequencing transcriptomics data and corresponding clinical information were retrieved for a total of 304 cervical cancer tissues and another 32 cancer types from the TCGA database (https://tcga-data.nci.nih.gov/tcga/ (accessed on 3 November 2020)). We excluded samples whose overall survival (OS) or survival status were not available. We also obtained GTF files from Ensembl (http://asia.ensembl.org (accessed on 3 November 2020)) for annotation of the mRNA. Besides, 24 normal and 28 cervical cancer samples (GSE63514) based on the GPL570 (Affymetrix Human Genome U133 Plus 2.0 Array) platform, as well as 300 early cervical cancer tissues (GSE44001) based on the GPL14951 (Illumina HumanHT-12 WG-DASL V4.0 R2 expression beadchip) platform were, respectively, collected from the GEO database (http://www.ncbi.nlm.nih.gov/geo (accessed on 3 May 2021)).

The differentially expressed genes (DEGs) were identified using the *R* package *limma*. The volcano plot of DEGs was plotted and visualized with the *R* package *ggplot2*. Meanwhile, the overlapping genes of DEGs and metabolism-related gene sets were presented using the Venn diagram online tool (http://www.bioinformatics.com.cn/static/others/jvenn/example.html (accessed on 4 May 2021)).

### 2.2. Functional Enrichment Analysis

Gene ontology (GO) analysis was performed using the R package *clusterProfiler* based on the DEGs between high- and low-score groups to speculate the possible function terms. The *p* values were adjusted via the BH method and an adjusted *p* value < 0.05 was considered statistically significant. Herein, we displayed some significant enrichment outcomes in the aspect of the biological process (BP). Furthermore, we also evaluate the enrichment levels of 50 hallmark pathways using R packages *GSVA* and *msigdbr*. Based on the GSVA score, we conducted the differential analysis for these hallmark pathways with the R package *limma* between high- and low-score groups. In addition, we also resorted to the online tool EMTome (www.emtome.org (accessed on 19 May 2021)) to obtain the enriched networks of five metabolism-related hub genes.

### 2.3. Construction of the Metabolism-Related Risk Score Signature

The association of the 211 overlapped genes with clinical outcomes of cervical cancer patients was analyzed via univariate Cox regression analysis. Those genes with a *p* value less than 0.05 were selected for further multivariate Cox regression analysis. In this manner, we identified five hub genes that had independent prognostic values. In this step, the *R* packages *plyr* and *survival* were applied. Then we developed the risk score signature consisting of the five hub genes, and the total risk score of this biosignature was calculated as the following formula: Risk Score = $\sum_{i=1}^{N}\beta_{i}\cdot Gene_{i}$, where *N* is the number of hub metabolism-related genes, *β_i_* refers to the regression coefficient and *Gene_i_* represents the expression level of each gene identified by multivariate Cox analysis. The cut-off for this risk model was defined as its median value. Subsequently, the patients were classified into high-risk and low-risk groups according to the median threshold. The ROC curve was plotted via the *R* package *timeROC* to assess the predictive potential of this signature for overall survival. The Kaplan-Meier survival curve was also conducted to evaluate the difference in patient prognosis of two subgroups using the *R* packages *survival* and *survminer.*

### 2.4. Predictive Power of This Signature in the Validation Cohort and Pan-Cancer Cohorts

The predictive performance of the risk score derived from the set of five metabolic genes was evaluated in the training cohort, as well as the validation cohorts. The multivariate Cox regression analysis demonstrated that the risk score served as an independent prognostic factor and, subsequently, we constructed an OS-related nomogram with this score using the *R* package *rms.* Furthermore, the C-index of this model was measured and the result suggested that the signature had reliable predictive power. Moreover, the calibration plots of this nomogram were presented to measure its predictive accuracy in comparison to the actual curve of the survival time.

The expression profile of early cervical cancer patients in GSE44001 was retrieved as a testing cohort to validate the model externally. The Kaplan-Meier analysis was used to explore the universality of this signature, while the ROC curve was also plotted via the *R* package *timeROC*. Moreover, we validated the predictive power of this model in other cancer types. We performed a pan-cancer analysis in 33 cancer types from the TCGA project by univariate Cox regression analysis, taking advantage of this risk score. The *R* packages *survival* and *plyr* were applied in this step.

### 2.5. Measurement of Tumor-Infiltrating Immune Cells and the Potential Response of Patients for Immunotherapy

An integrated list of representative marker genes of tumor-infiltrating immune cell types was acquired from Charoentong’s research, which involved a total of 366 microarrays of immune cells summarized from 37 previous studies [17]. To evaluate the infiltration of immune cells, we conducted the single-sample gene set enrichment analysis (ssGSEA) algorithm using these marker gene sets of different immune cells with the *R* package *GSVA*, which could measure the normalized enrichment score of multiple immune cell types. The tumor abundance of these immune cells between high-and low-risk groups was also plotted using the *ggplot2* package. In the meantime, we downloaded the enriched results from ImmuCellAI, which estimated the abundance of 24 immune cells in the TCGA-CESC cohort [18]. The immune network of 24 ImmuCellAI cell types in the TCGA cohort was illustrated by the *R* packages *reshape2*, *corrplot* and *igraph*. In this Circos plot, we shed light on the correlation among these cell types and their survival impact by Spearman correlation analyses and univariate Cox regression analyses. Correlation between the established signature and these immune cells was also calculated by Spearman analyses using the *R* software.

Kaplan-Meier analysis was carried out to clarify the relationship between the prognosis of patients with similar expression levels of immune checkpoints and the established signature. The *R* packages *survival* and *survminer* were utilized to draw the plot and conduct multiple comparisons between different survival curves.

### 2.6. Tissue Microarray and Immunohistochemical (IHC) Staining

The study was approved by the Institutional Ethics Committee of Shanghai General Hospital. Cervical cancer (*n* = 32) and normal cervix (*n* = 32) tissue microarrays were purchased from Shanghai Zuocheng Biotech (Shanghai, China). We performed the IHC analysis as previously described [19]. The antibodies used in the study were listed as follows: anti-ALOX12B rabbit polyclonal antibody (NBP1-89409, Novus Biologicals, Colorado, CO, USA), anti-FAR2 rabbit polyclonal antibody (NBP1-90435, Novus Biologicals, Colorado, CO, USA), anti-F5 rabbit polyclonal antibody (20963-1-AP, Proteintech, Wuhan, China), anti-CA9 rabbit polyclonal antibody (11071-1-AP, Proteintech, Wuhan, China), anti-TDO2 rabbit polyclonal antibody (15880-1-AP, Proteintech, Wuhan, China), anti-CD4 mouse monoclonal antibody (67786-1-Ig, Proteintech, Wuhan, China), anti-CD8 mouse monoclonal antibody (66868-1-Ig, Proteintech, Wuhan, China), anti-CD57 rabbit polyclonal antibody (19401-1-AP, Proteintech, Wuhan, China) and anti-CD68 mouse monoclonal antibody (66231-2-Ig, Proteintech, Wuhan, China). The immunoreactivity score (IRS) was used to evaluate the expression level of each protein. The staining intensity was scored as: negative = 0, weak = 1, moderate = 2 and strong = 3. The staining extent was scored as: 0 (no positive cells), 1 (≤25% positive cells), 2 (26–49% positive cells), 3 (50–74% positive cells) and 4 (≥75% positive cells). IRS = extent score × intensity score. The numbers of CD4^+^ cells, CD8^+^ cells, CD57^+^ cells and CD68^+^ cells at the tumor site were counted under five randomly selected microscopic fields.

### 2.7. Scoring of Immune Cell Infiltration

We performed the scoring of immune cell infiltration (CD4^+^ cells, CD8^+^ cells, CD57^+^ cells and CD68^+^ cells) according to the methods used in the previous study [20]. Briefly, stained samples were assessed and scored on a five-point scale for the infiltration level of cells into epithelial or stromal areas, with regard to the range of infiltration, as the following scale: no positive events found on slide = 1, rare positive events observed = 2, low density of infiltration = 3, medium density of infiltration = 4 and high density of infiltration = 5.

### 2.8. Statistical Analysis

All of the statistical process was completed within the *R* software (version 4.0.4). The Wilcoxon test was applied to compare between two groups, while the Kruskal-Wallis test was used to compare among more than two groups. The Kaplan-Meier plot was performed to present survival curves for different groups, and the log-rank test was employed to evaluate the significance of statistical differences. Spearman analysis was carried out to determine the correlation coefficient. For all the analyses above, a two-tailed *p* < 0.05 was considered as statistically significant.

## 3. Results

### 3.1. Construction of a Metabolism-Related Risk Score Signature in Cervical Cancer

**Figure 1:**
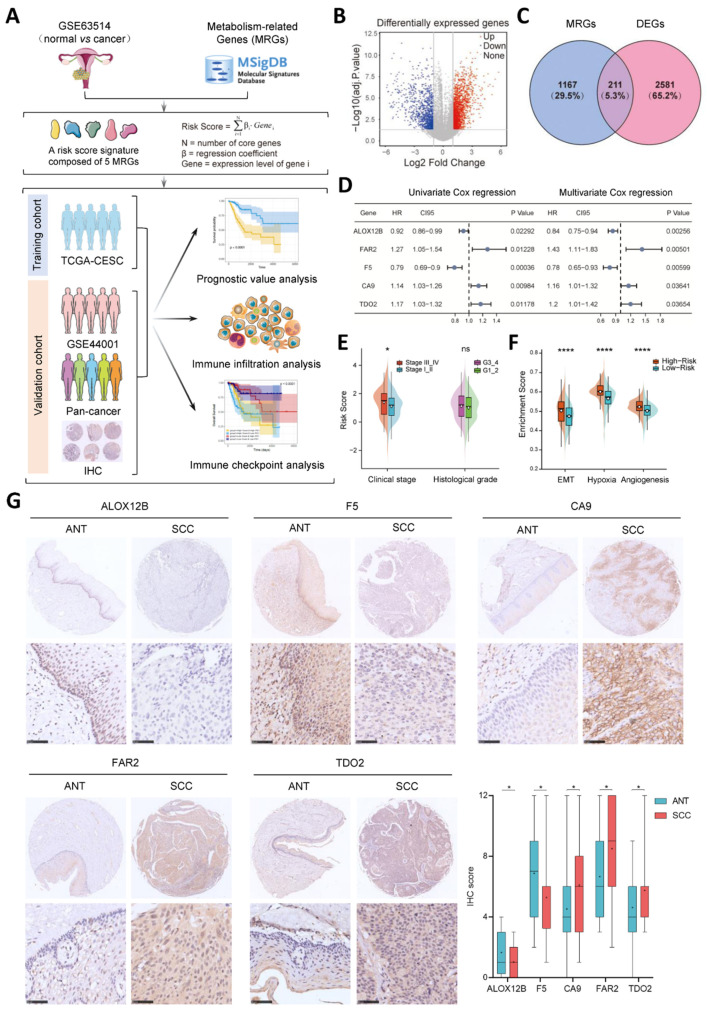
Construction of a metabolism-related risk score signature in cervical cancer. (**A**) Schematic diagram of this study design. (**B**,**C**) Identification of differentially expressed metabolism-related genes (DEGs) between tumor and normal samples using GEO dataset (GSE63514) and annotation of GPL570 platform. The volcano plot (**B**) and Venn diagram (**C**) are shown. (**D**) The uni- and multi-variate Cox regression analysis results of the five metabolism-related hub genes in TCGA-CESC cohort. (**E**,**F**) The association between risk score and clinicopathological characters as well as enrichment scores of specific gene sets, including EMT, angiogenesis and hypoxia, of patients in TCGA cohort. The statistical difference of two groups was compared through the Wilcoxon test. * *p* < 0.05; **** *p* < 0.0001. (**G**) Representative immunostaining pictures of the five hub genes (ALOX12B, FAR2, F5, CA9 and TDO2) in tumor and normal tissues. Scale bar = 50 μm. The protein levels were plotted as a boxplot. * *p* < 0.05. MRGs, metabolism-related genes; ANT, adjacent non-tumor tissue; SCC, squamous cell carcinoma.

The whole flow diagram of this study is presented in Figure 1A. To screen differentially expressed metabolism-related genes in cervical cancer, we extracted the expression profiling data of a cohort from GSE63514 and collected a panel of 1378 metabolism-related genes from MSigDB. As demonstrated in Figure 1B,C, the volcano plot visualized the DEGs and a total of 211 metabolism-related DEGs showed significant dysregulation in the GEO dataset. After being intersected with the expression profile of the TCGA-CESC cohort, 206 overlapping genes were taken into account for further study.

The univariate Cox regression was implemented to examine the prognostic value of metabolism-related DEGs based on the transcriptome data from the TCGA-CESC project. Those DEGs whose *p* < 0.05 subsequently underwent a multivariate Cox regression. Eventually, five genes (ALOX12B, CA9, F5, FAR2 and TDO2) with significant regression coefficients were incorporated to set up a prognostic risk model (Figure 1D and Supplementary Table S1). The risk score based on the set of the five metabolic genes was calculated with the following formula: risk score = −0.1083206 × ALOX12B + 0.1375361 × CA9 − 0.3202668 × F5 + 0.2241991 × FAR2 + 0.2649977 × TDO2.

The relationship between the risk score and clinicopathological characteristics was explored. We found that patients with a more advanced clinical stage displayed higher risk scores (*p* = 0.0244), whereas there was no significant association between the signature and the histological grade of TCGA-CESC patients (Figure 1E). There were also statistically significant differences between the high- and low-risk groups in terms of the enrichment score of gene sets correlated with hypoxia, EMT and angiogenesis (*p* < 0.0001, Figure 1F). Among the five genes, the expression of ALOX12B and F5 was decreased, while that of CA9, FAR2 and TDO2 was relatively elevated in cervical cancer samples, as indicated by the IHC results (Figure 1G).

### 3.2. Verification of the Metabolism-Related Risk Score Signature

**Figure 2:**
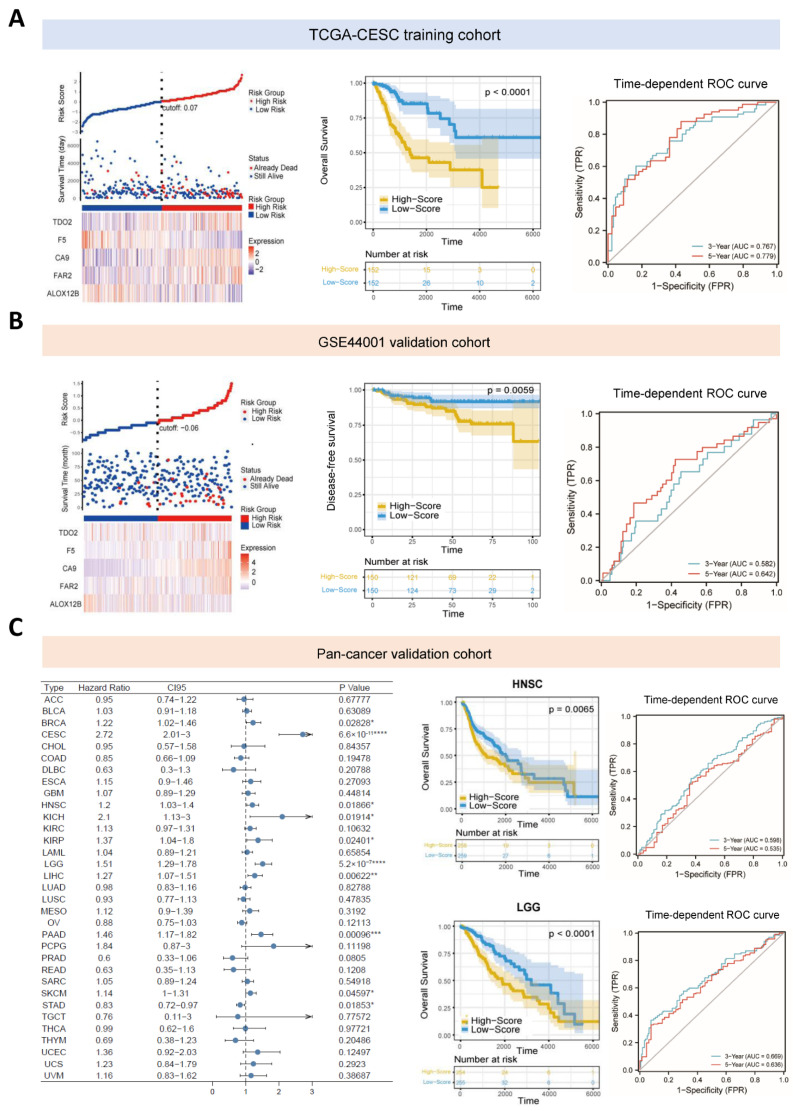
Verification of the metabolism-related risk score signature. (**A**) The left panel shows the risk curve and scatter plot of each sample in TCGA-CESC project reordered by the risk score, and the heatmap of expression profiles of the five hub genes. The middle panel displays the results of Kaplan-Meier analysis. The 3- and 5-year ROC curves curves of this optimal model (**the right panel**) revealed the AUC values. (**B**) The risk curve, heatmap of expression profiles (**the left panel**), results of Kaplan-Meier analysis (the middle panel) and ROC curves at 3 and 5 years (**the right panel**) of patients in GSE44001 cohort. (**C**) Forest plot of the univariate Cox regression analyses results of this risk score signature in all 33 types of cancer from TCGA database (**the left panel**). The Kaplan-Meier survival analyses and ROC curves of TCGA-HNSC and TCGA-LGG cohorts were plotted on the right panel. AUC, area under curve; ROC, receiver operating characteristic. * *p* < 0.05; ** *p* < 0.01; *** *p* < 0.001, **** *p* < 0.0001.

Then we calculated the metabolism-related risk score of all the CESC patients according to the formula and re-divided them into high-risk and low-risk groups based on the median cut-off value. Evidently, the high-risk group comprised more death cases than the low-risk group, and the differential expression levels of these genes conformed to their prognostic impact (Figure 2A). A Kaplan-Meier survival analysis revealed that patients in the high-risk group exhibited significantly worse clinical outcomes (*p* < 0.0001, Figure 2A). The time-dependent ROC curve indicated that the risk score had a relatively higher accuracy in predicting the 3-year (AUC = 0.767, NPV = 0.846, PPV = 0.583) and 5-year OS (AUC = 0.779, NPV = 0.901, PPV = 0.514) (Figure 2A). A prognostic nomogram was also established based on the predictive indicator of the risk score, which proved to be an independent prognostic factor in CESC patients (Figure S1A). The c-index of this model was 0.742 and the calibration curve demonstrated that the nomogram-predicted overall survival was close to the actual values of the 1-, 3- and 5-year survival rates (Figure S1B). The decision curve analysis (DCA) further indicated that the risk score benefited patients with CESC in clinical practice (Figure S1C).

An independent GEO dataset (GSE44001) was used to validate the aforementioned risk score. Three hundred early cervical cancer patients were categorized into low- and high-risk groups based on the median cut-off value, and the heatmap of expression levels of the five hub genes validated their differential distribution between the two subgroups (Figure 2B). Similarly, the Kaplan-Meier survival analysis revealed that patients in the high-risk group displayed significantly worse clinical outcomes (*p* = 0.0059, Figure 2B), and the time-dependent ROC curve also suggested that the risk score had a relatively higher reliability in predicting the 3-year (AUC = 0.582, NPV = 0.938, PPV = 0.13) and 5-year DFS (AUC = 0.642, NPV = 0.921, PPV = 0.238) (Figure 2B).

Meanwhile, the pan-cancer validation analysis in the other 32 cancer types of the TCGA project revealed that the risk score composed of the five metabolism-related genes could successfully discriminate between patients with better or worse clinical outcomes in the other eight cancer types (Figure 2C), such as breast invasive carcinoma (BRCA), head and neck squamous cell carcinoma (HNSC), brain lower grade glioma (LGG), liver hepatocellular carcinoma (LIHC), pancreatic adenocarcinoma (PAAD), etc. Therefore, these results suggested that the metabolism-based risk score might be a promising prognostic classifier, which could be applied to predict the clinical outcome of patients with various types of malignancy.

### 3.3. Functional Enrichment Analysis of the Metabolism-Related Risk Score Signature

**Figure 3:**
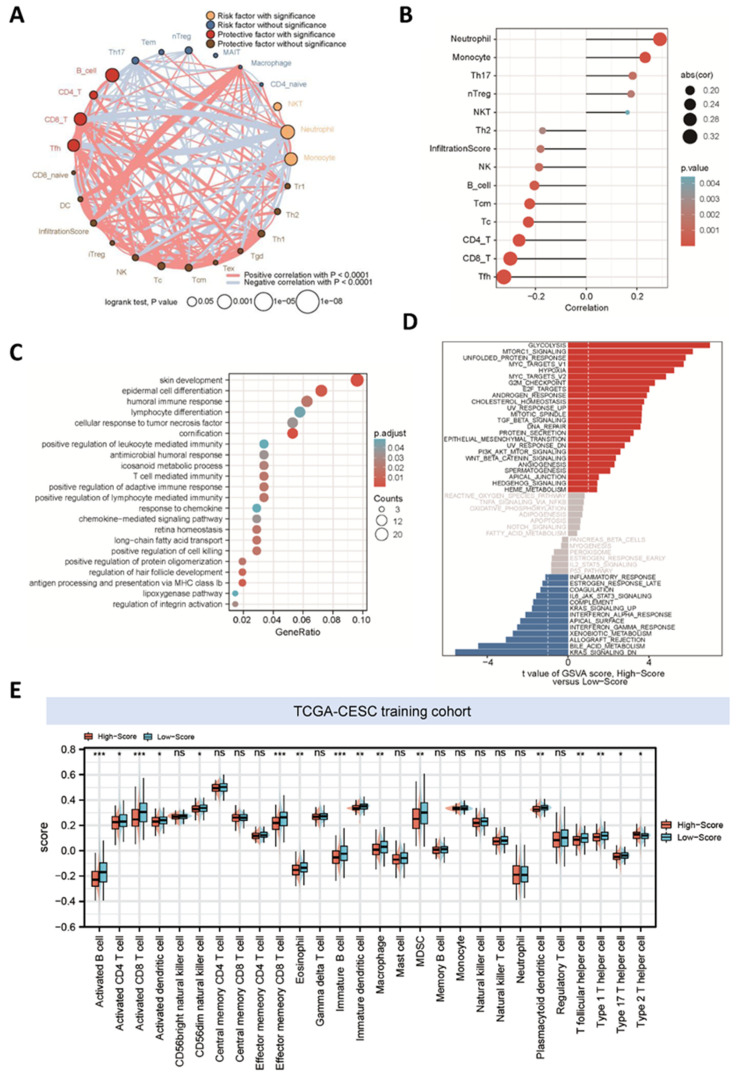
Functional enrichment analysis of the metabolism-related risk score signature. (**A**) Immune network of the 24 ImmuCellAI cell types in the TCGA cohort. The size of each cell was calculated by the formula log10 (*p*-values of univariate Cox regression analyses). The color of each cell was used to represent the different survival impact of these cell types. The thickness of the lines estimated by Spearman correlation analyses depicted the strength of correlation between diverse cell types. Red represents positive correlation whereas negative is in blue. (**B**) Correlation between these cell types and our established risk score in the TCGA cohort. Spearman analyses were applied to calculate the correlation coefficients and *p*-value < 0.05 was enrolled. (**C**) Gene ontology (GO) enrichment analysis of the differentially expressed genes between high- and low-risk groups in the TCGA-CESC cohort. Adjusted *p*-value < 0.05 was considered statistically significant. (**D**) GSVA analysis of hallmark pathways in the TCGA cohort was performed. Differential analysis of GSVA score between high- and low-risk groups was displayed. (**E**) Patients in the high-risk group were associated with lower infiltrating density of most cell types according to Charoentong’s research. * *p* < 0.05; ** *p* < 0.01; *** *p* < 0.001.

According to the estimated infiltration levels of 24 immune cells from the ImmuCellAI database, we visualized the immune landscape of patients in the TCGA-CESC cohort via an immune network (Figure 3A). Among the 24 immune cells, B cells, Tfh cells, CD4 T cells and CD8 T cells were protective factors with significance, while neutrophils and monocytes were significant risk factors. Our established signature showed a positive correlation with these risk factors but it was negatively correlated with the protective immune cells (Figure 3B).

GO and GSEA analyses were conducted to shed light on the possible function of these DEGs and the underlying mechanism. As shown in Figure 3C, significantly enriched GO terms involved ‘T cell mediated immunity’, ‘humoral immune response’ and ‘antigen processing and presentation via MHC class Ib’. In addition, GSEA analysis suggested a series of enriched hallmark pathways that were activated in the high-score group compared with the low-score group (Figure 3D). For instance, the upregulated pathways involved ‘glycolysis’, ‘DNA repair’, ‘epithelial-mesenchymal transition’ and ‘TGFβ signaling’, while the suppressed pathways included ‘inflammatory response’, ‘IL6-JAK-STAT3 signaling’ and ‘IFNα response’. These results suggested that the risk score might play a crucial role in the regulation of the immune response.

Furthermore, we also obtained the enrichment terms of the five metabolism-related hub genes via the online tool EMTome and the enriched networks are shown in Supplemental Figure S2. Moreover, we quantified the enrichment score of the metabolic-related pathways, and plotted their abundance in high- and low-risk groups (Supplemental Figure S3A,B).

### 3.4. Correlation between the Metabolism-Related Risk Score and Immune Landscape

The normalized enrichment scores (NES) of different immune cells in the TCGA-CESC project were calculated based on the gene expression profiles via the ssGSEA algorithm (Figure 3E). We found that specimens in the high-risk group were conferred a significantly lower infiltrating density of various immune cells, such as activated CD4 T cells, activated CD8 T cells, macrophages and CD56dim natural killer cells. In addition, we performed Spearman correlation analyses and found that the risk score and five hub genes were closely linked to the immune landscape (Supplementary Figure S4).

**Figure 4:**
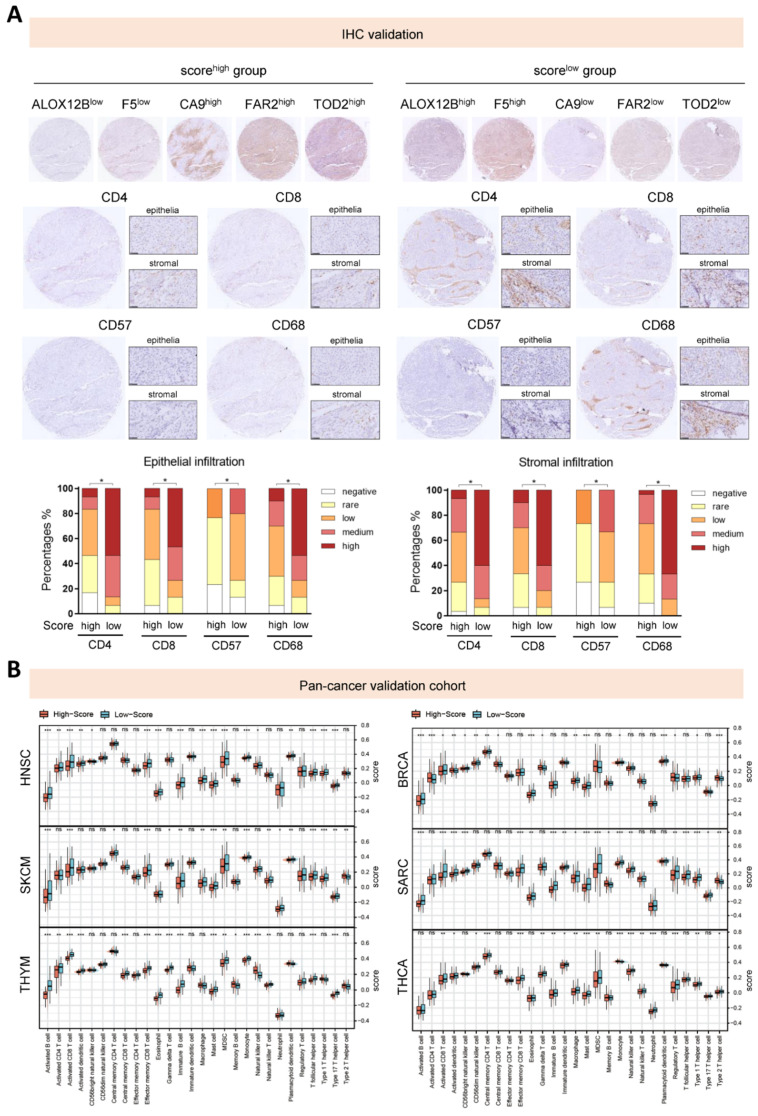
Correlation between the metabolism-related risk score and immune landscape. (**A**) Representative immunostaining pictures of the five hub genes and four cell types (CD4+, CD8+, CD57+ and CD68+ cells). The upper panel comprises images of five hub genes, images of four cell types were in the middle panel. Scale bar: 50 μm. The lower panel illustrates the infiltration scores of tumor-infiltrating CD4+, CD8+, CD57+ and CD68+ cells in the epithelial or stromal cell compartments. * *p* < 0.05. (**B**) Differences in the infiltration levels of 28 immune cells between high- and low-score groups in the pan-cancer validation cohorts. * *p* < 0.05; ** *p* < 0.01; *** *p* < 0.001.

To verify the difference in infiltration levels of immune cells, we stained histology sections for immune markers including CD4, CD8, CD57 and CD68, and scored for the extent of infiltration both in the epithelial and stromal tumor compartments based on the density of positive staining for immune cell populations (Figure 4A). The IHC results revealed that in the high-risk group, CD4 T cells, CD8 T cells, macrophages and NK cells exhibited consistently significantly lower densities of infiltration both in the stromal and epithelial content. The outcome suggested that detectable differences in immune cell recruitment was correlated with our established risk score.

In the pan-cancer cohorts, our conclusion was also validated in a number of cancer types, as shown in Figure 4B and Supplementary Figure S5. It was obvious that patients with higher levels of our established risk score tended to exhibit lower infiltration of diverse immune cells across several cancer types.

### 3.5. Prognostic Value of the Established Signature in Cervical Cancer Patients for Immunotherapies

**Figure 5:**
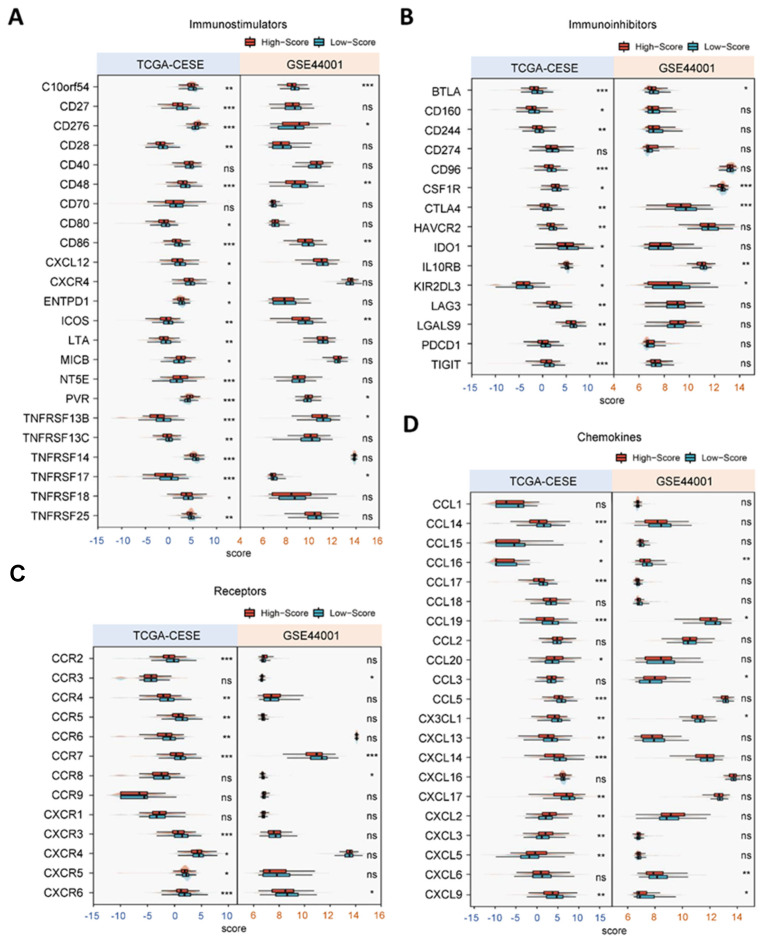
Different expression patterns of immunostimulators (**A**), immunoinhibitors (**B**), receptors (**C**) and chemokines (**D**) between high- and low-score groups in TCGA training cohort and GSE44001 validation cohort. * *p* < 0.05; ** *p* < 0.01; *** *p* < 0.001.

Meanwhile, we retrieved the list of genes encoding immunostimulators, immunoinhibitors, chemokines and receptors from the TISIDB website [21]. Notably, the high- and low-risk groups displayed distinct expression patterns of these immune-related molecules, which also depicted their disparity in the immune landscape (Figure 5). For instance, the expression levels of most immunostimulators, involving ICOS and TNFRSF members, were augmented in the low-risk group rather than the high-risk group (Figure 5A).

**Figure 6:**
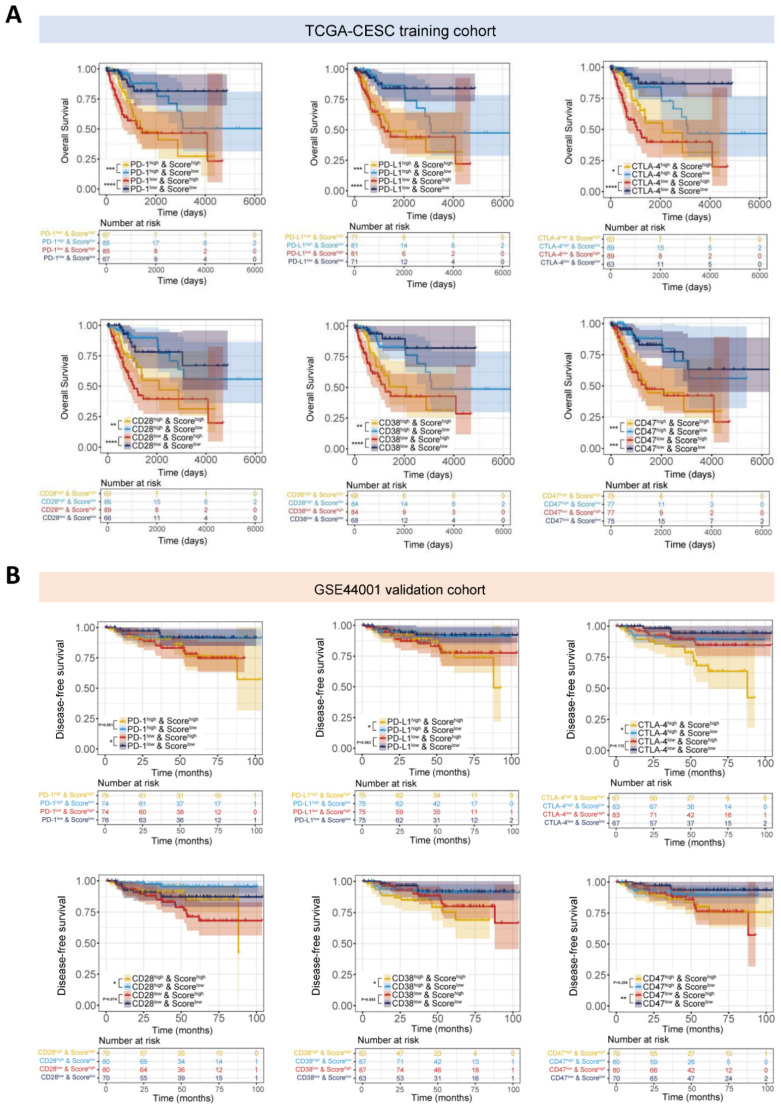
The potential of the established signature in predicting the immunotherapeutic benefits of cervical cancer patients. (**A**) Kaplan-Meier curves for patients in the TCGA-CESC cohort stratified by the risk score and expressions of immune checkpoints, such as PD1, PD-L1, CTLA-4, CD28, CD38 and CD47. * *p* < 0.05; ** *p* < 0.01; *** *p* < 0.001; **** *p* < 0.0001. (**B**) Kaplan-Meier curves for patients in the GSE44001 validation cohort stratified by the risk score and expressions of immune checkpoints, such as PD1, PD-L1, CTLA-4, CD28, CD38 and CD47. * *p* < 0.05; ** *p* < 0.01.

Furthermore, we stratified patients in the training cohort, as well as the validation cohort, with our established signature based on the expression levels of different immune checkpoints (ICPs), and found that a higher risk score indicated a worse prognosis in patients with similar levels of ICPs (Figure 6). Consistently, we observed that patients with a lower risk score on the basis of similar expression of ICPs experienced a more favorable clinical outcome. For example, patients with a high risk score and high PD-1 displayed a shortened OS or DFS time compared to those with a low risk score and high PD-1 (*p* < 0.001). Similar survival patterns could be also observed using the risk score and PD-L1, CTLA-4, CD47, CD38, CD28 and BTLA (Figure 6 and Supplementary Figure S6). These observations suggested that the risk score might serve as a predictive biomarker of treatment response to immunotherapies.

## 4. Discussion

Previous studies have established metabolism-related signatures for the survival prediction of patients with several types of cancer [14,22]. Herein, we developed a novel risk score signature based on the expression of five metabolism-related hub genes and evaluated its prognostic value in cervical cancer patients. This prognostic score model was confirmed in an independent cervical cancer validation cohort and pan-cancer cohorts. Furthermore, we validated the correlation between this risk score and immunologic features, which might potentially improve the accuracy of predicting the clinical outcome of patients, in combination with the conventional clinical staging. To our knowledge, this is the first study to establish a metabolism-related risk score model in cervical cancer patients, which could also be testified in various kinds of cancers simultaneously.

The TME is a complicated system which undergoes dynamic changes [23]. The complex components of the TME nurture the surrounding environment that is essential for tumor growth. More and more studies have revealed that specific metabolic patterns of the TME potentiate tumor progression or treatment resistance [24,25,26]. Under specific conditions such as hypoxia, metabolic reprogramming occurs to enhance cellular proliferation, and cancer cells have been proven to facilitate the metabolism of reactive oxygen species, lactate, lipids, glutamine and glucose, as well as amino acids [27]. For instance, rather than the oxidative phosphorylation conducted by normal cells, cancer cells are inclined to adopt lactate metabolism and glycolysis [28]. Besides, atypical lipid metabolism has also been linked to tumor recurrence and CD8^+^ T cell exhaustion, giving rise to post-chemotherapy evasion of immune surveillance [29,30]. Metabolic reprogramming and redox imbalances were also revealed to mediate the development and maintenance of dormant cancer cells in various malignancies, which was caused by endoplasmic reticulum (ER) stress responses and oxidative stress [31]. As a result, exploring the specific metabolic disorders and determining several metabolism-related genes that were implicated in tumorigenesis would help to predict the prognosis and therapeutic responsiveness. To this end, we developed and validated a novel metabolism-related risk score signature consisting of five metabolism-related hub genes to predict the clinical outcomes of cervical cancer patients. The AUC of the ROC curves of the TCGA cohort, based on this risk score signature model, was higher than 0.76 at the 3- and 5-year OS. Importantly, our results pinpointed that the high-risk group was characterized by a lower extent of immune infiltration. Bioinformatic enrichment analyses also identified several immune-related signaling pathways as potentially relevant pathways between the high- and low-score groups. Considering that the immunosuppressive microenvironment could facilitate tumor progression, the high-risk score patients were presumed to bear a higher tumor burden.

Biomarker-based patient stratification has gained much attention, which calls for more accurate evaluation of these molecular properties. Especially for those patients who receive immunotherapy, the comprehensive analysis of the risk score, as well as immune checkpoint expression, could hopefully foretell the reactivity of these patients and therefore screen out the appropriate patients for immunotherapy [32,33,34]. Immune checkpoints expressed on cancer cells or cancer-associated immune cells have drawn substantial attention as promising treatment targets nowadays [35]. A mounting number of studies have attempted to develop immune-based biomarker signatures to depict the survival rate and tumor progression of patients [35,36,37,38,39]. In this study, we analyzed the expression levels of these immune checkpoints in the context of cervical cancer. As Figure 6 shows, patients in the high-risk group exhibited shorter overall survival or disease-free survival on the condition that they had similar expression levels of ICPs. This exploration may potentially guide a more personalized treatment for immunotherapies.

As a member of our risk score model, tryptophan 2,3-dioxygenase 2 (TDO2) catalyzes the commitment step of the KYN metabolic process, which subsequently activates the AhR and contributes to an immunosuppressive TME, and supports the cancer immune escape [40,41]. FAR2, namely the fatty acyl-CoA reductase 2, has been found to be localized in the peroxisome and participate in the first step of wax biosynthesis [42]. Previous studies have shown that overexpression of FAR2 induced the upregulation of platelet-activating factor (PAF) and profibrotic cytokine TGF-β in a mouse mesangial cell line. Another research revealed that FAR2 mediated the de novo synthesis of PAF, a potent inflammatory mediator activating platelets, eosinophils, neutrophils and macrophages, in vitro [42,43]. As for carbonic anhydrase 9 (CA9), a pH-regulating transmembrane protein which is overexpressed in solid tumors, it was proven to repress the mitochondrial biogenesis, favor the Warburg phenotype and activate glycolysis [44,45]. CA9 equilibrates among hypoxia, iron metabolism, and redox regulation in tumor cells [46]. Beyond that, CA9 also upregulated amino acid transporters to increase the intracellular content. Ectopic expression of CA9 is a biomarker of poor prognosis in breast cancer, tongue squamous cell carcinoma, and pancreatic and lung cancers [47,48,49].

On the other side, high expression of coagulation factor 5 (F5) was found to be associated with improved overall survival of patients with breast cancers [50]. Therefore, tumor-derived F5 appears to be beneficial to patient survival, which is compatible with the tumor suppressor function proposed by our study. Consistently, the ectopic expression of F5 in breast tumors could also represent a more infiltrated microenvironment with both lymphoid and myeloid cells, such as T cells, NK cells and macrophages, according to a research conducted by Tinholt et al. [51]. ALOX12B encodes an enzyme involved in the conversion of arachidonic acid to 12R-hydroxyeicosatetraenoic acid, which has been proposed by Song et al. as a potential protective gene for the overall survival of patients with esophageal squamous cell carcinoma [52]. Egolf et al. identified ALOX12B as a driver gene of ferroptosis, a form of programmed tumor suppressive cell death featured by lipid peroxidation [53]. In the current study, we established the relationship between the metabolism-related gene signature constructed by these genes and the immune landscape of patients with cervical cancer, whereas the underlying mechanism of these hub genes still awaits to be unraveled.

Our study also has its limitations due to objective reasons. We analyzed the expression levels of metabolism-related hub genes in tumor specimens of the TCGA database, whereas we could not distinguish the cell origin of these hub genes. This means that our conclusion only represents the immunity pattern on the macro level. Further, single-cell sequencing data might better illustrate the complex association between tumor cells and the surrounding microenvironment. In the meantime, it has been a controversial issue whether the relatively small piece of tissues in TMAs could fully represent the characteristics of original tissues due to tumor heterogeneity, which might give rise to disparities in diagnosis. Since we affirmed our conclusion with an independent TMA cohort, the experimental results might also show some technical limitations to a certain extent.

## 5. Conclusions

In summary, our constructed metabolism-related prognostic model allows for a more accurate categorization of patients at different risk levels of cervical cancer. We also determined the infiltration of TILs, the expression pattern of immune-related molecules and the prognostic value of our signature for immunotherapies. Since this risk score signature achieves a good performance in predicting clinical outcomes, we genuinely expect that our study could provide an effective prognostic tool for the guidance of personalized treatment for cervical cancer patients.

## Data Availability

The data presented in this study are available in this article (and Supplementary Material).

## Supplementary Materials

The following supporting information can be downloaded at: https://www.mdpi.com/article/10.3390/cancers14102399/s1; Figure S1: Internal validation of the prognostic potential of the risk score signature; Figure S2: The results of functional enrichment analyses of the five metabolism-related hub genes; Figure S3: The association between the risk score and metabolism-related pathways; Figure S4: Spearman correlation analyses of the established risk score as well as five metabolism-related hub genes (ALOX12B, FAR2, F5, CA9 and TDO2) and tumor-infiltrating cells; Figure S5: Differences in the infiltration levels of 28 immune cells between high- and low-score groups in the pan-cancer validation cohorts (LUSC, UCEC and LAML); Figure S6: Kaplan-Meier curves for patients in the TCGA-CESC and GSE44001 cohorts stratified by the risk score and expressions of immune checkpoint BTLA; Table S1. Univariate and multivariate Cox regression proportional hazards model to analyze the correlation between the expression levels of 206 candidate genes and overall survival of patients with cervical cancers.

## Abbreviations

AUC, area under curve; DCA, decision curve analysis; DEG, differentially expressed gene; EMT, epithelial to mesenchymal transition; ICP, immune checkpoint; OS, overall survival; DFS, disease-free survival; NPV, negative predictive value; PPV, positive predictive value; ROC, receiver operating characteristic; TCGA, The Cancer Genome Atlas; TIL, tumor infiltrating lymphocyte; TME, tumor microenvironment.
