# Supplementary figures and images for: The Immunological Contribution of a Novel Metabolism-Related Signature to the Prognosis and Anti-Tumor Immunity in Cervical Cancer
Source: Cancers (Basel). 2022 May 13;14(10):2399. doi: 10.3390/cancers14102399 (PMC9139200; doi:10.3390/cancers14102399)

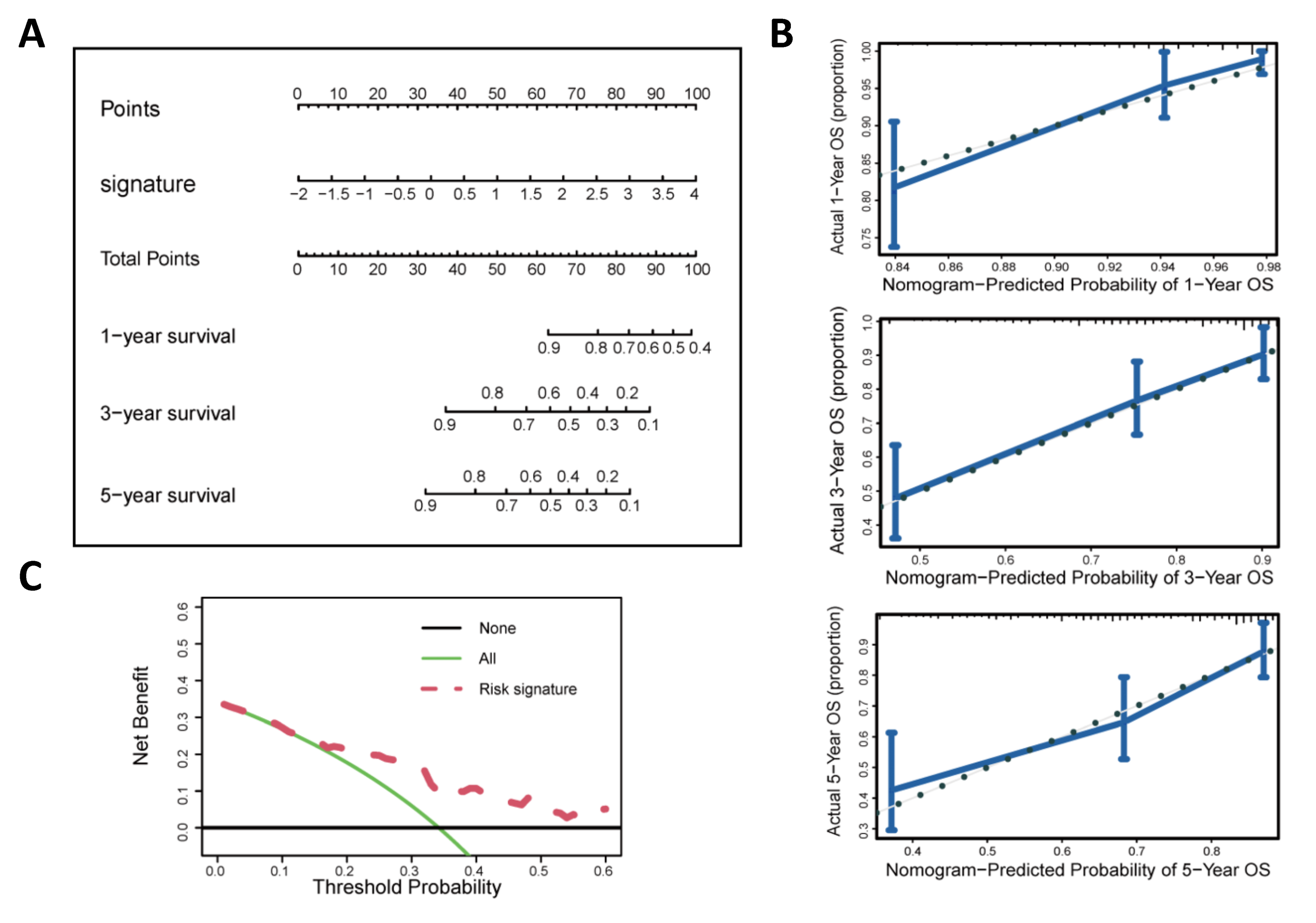

Supplement: Supplementary file 1 [file cancers-14-02399-s001.zip › Figure S1.tif]

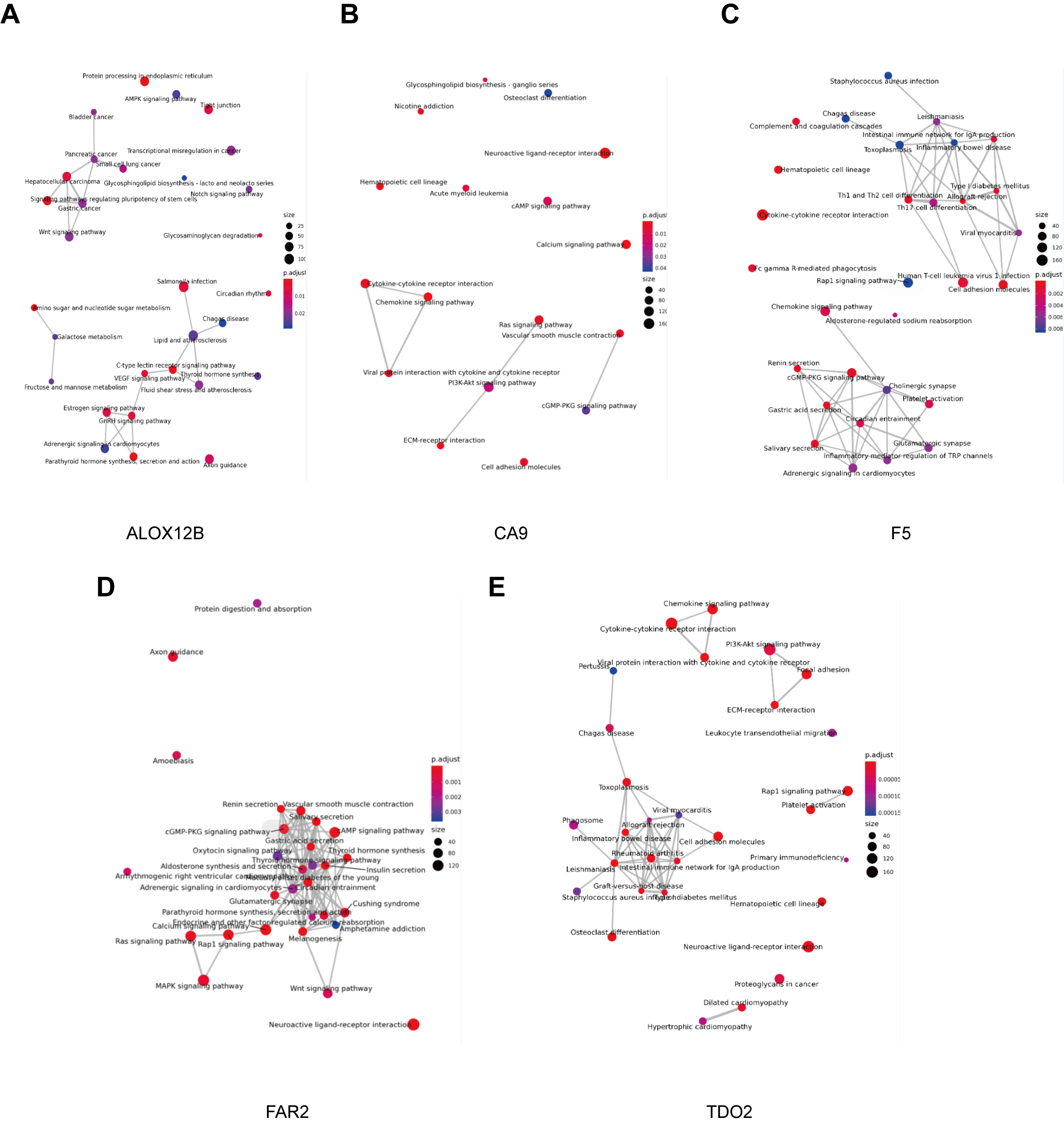

Supplement: Supplementary file 1 [file cancers-14-02399-s001.zip › Figure S2.tif]

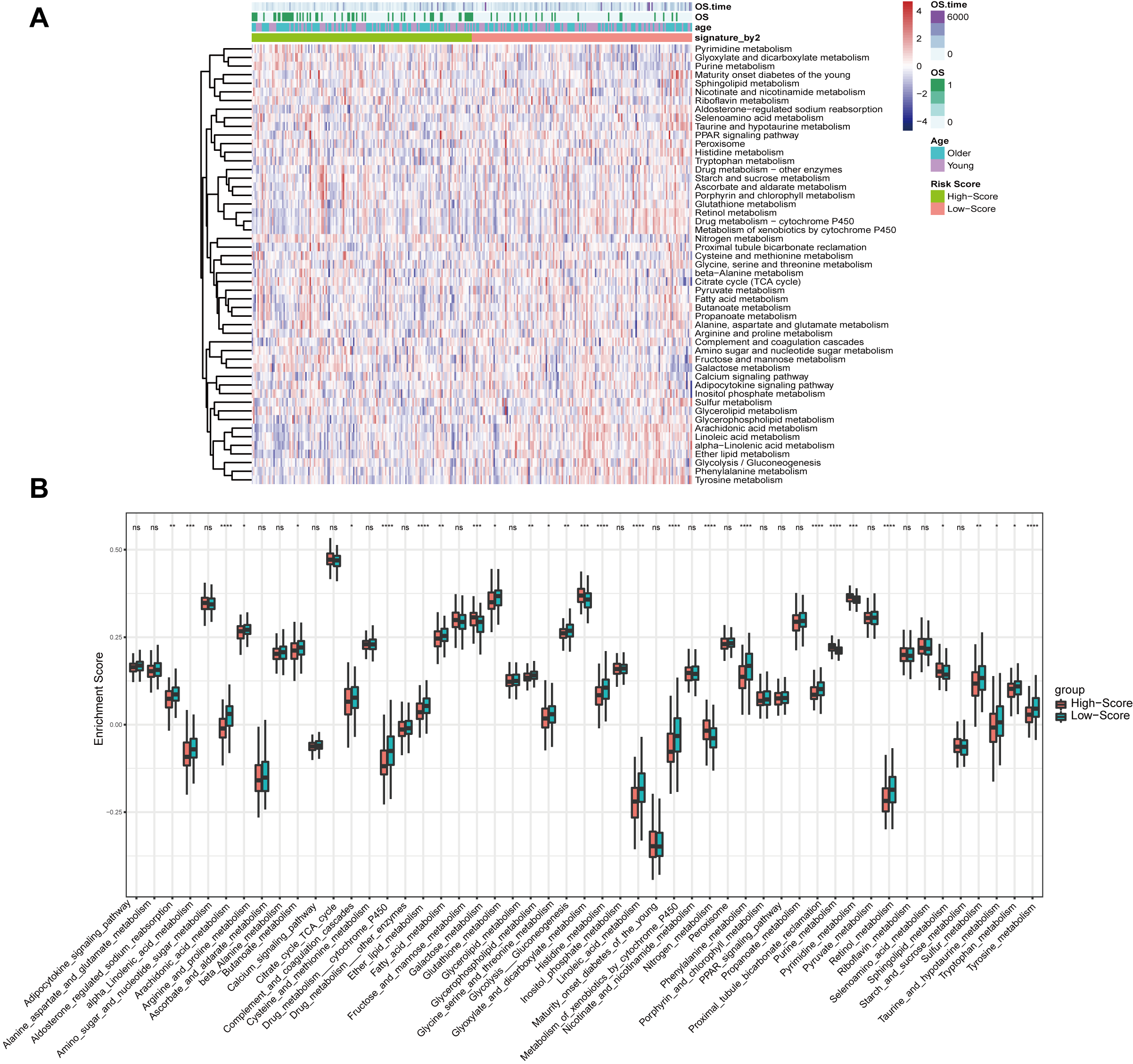

Supplement: Supplementary file 1 [file cancers-14-02399-s001.zip › Figure S3.tif]

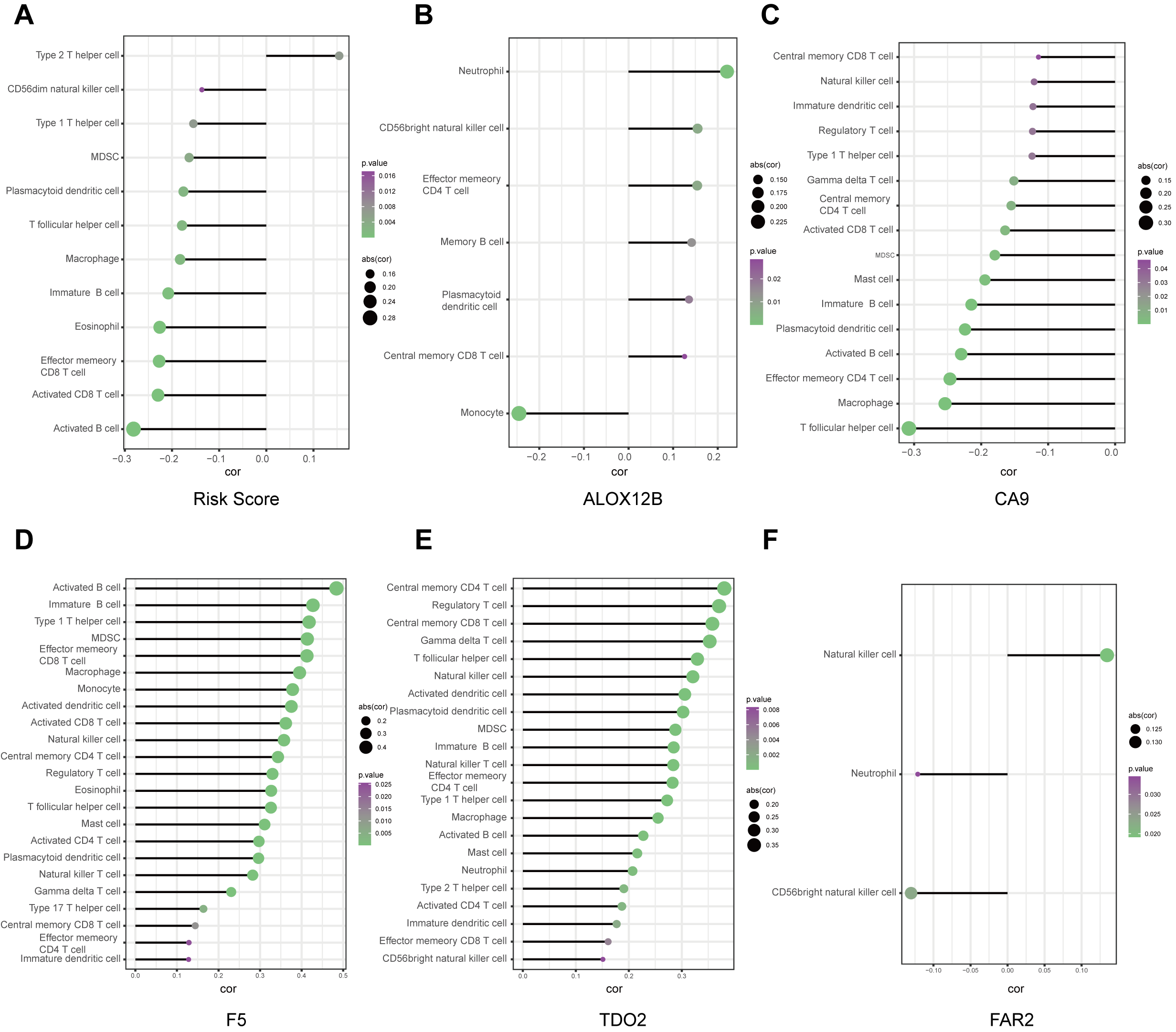

Supplement: Supplementary file 1 [file cancers-14-02399-s001.zip › Figure S4.tif]

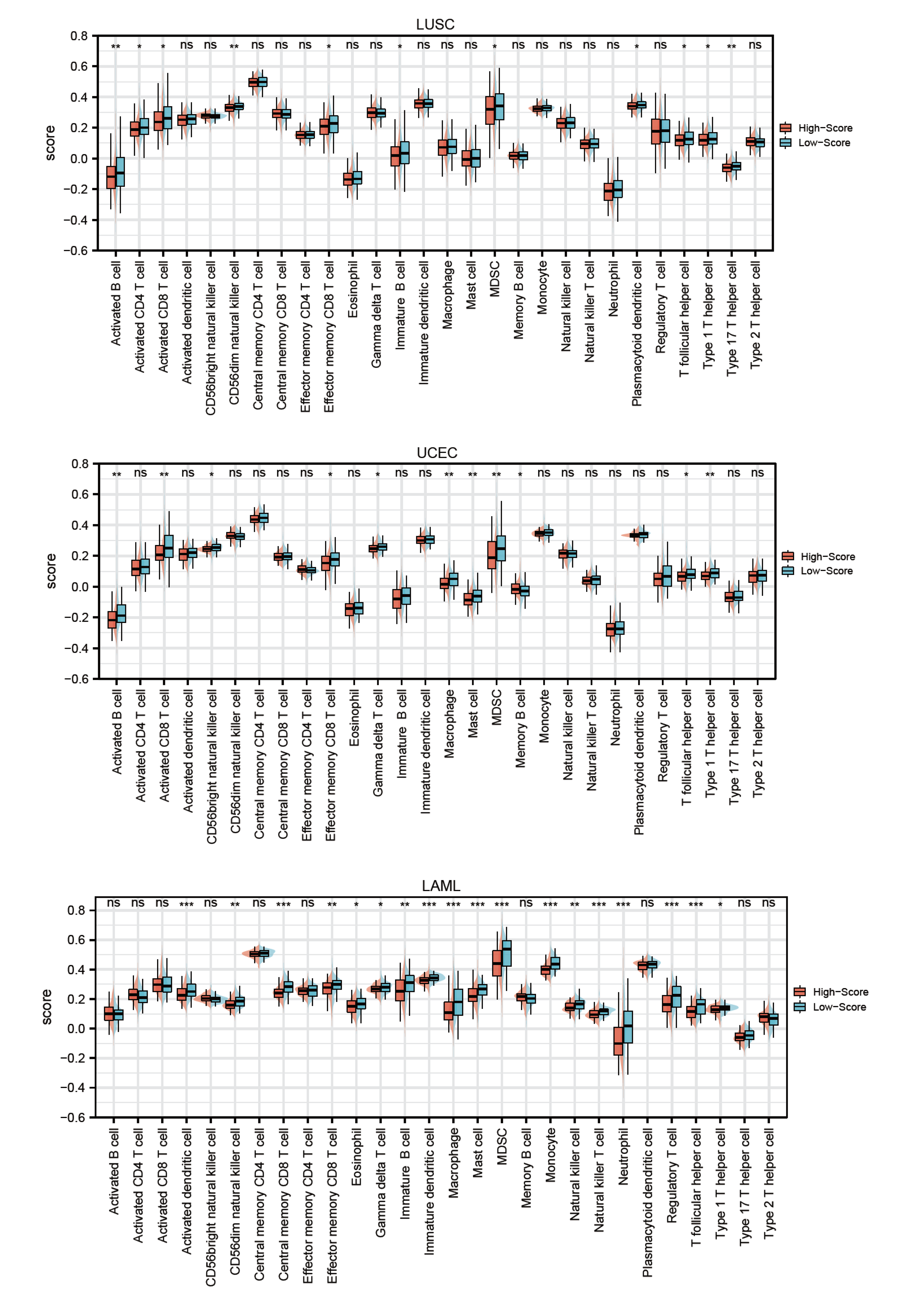

Supplement: Supplementary file 1 [file cancers-14-02399-s001.zip › Figure S5.tif]

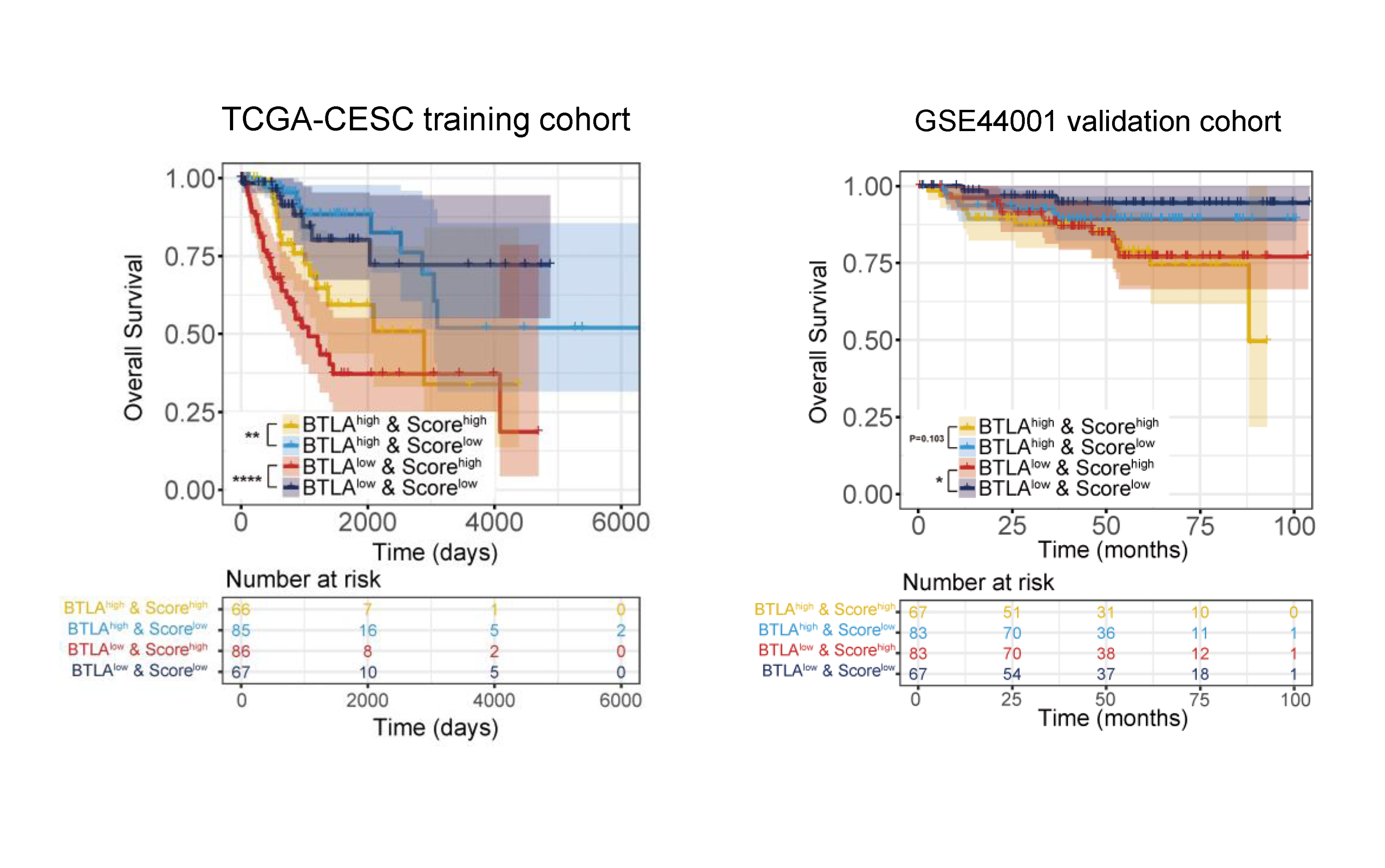

Supplement: Supplementary file 1 [file cancers-14-02399-s001.zip › Figure S6.tif]
