# Supplementary material for: The Immunological Contribution of a Novel Metabolism-Related Signature to the Prognosis and Anti-Tumor Immunity in Cervical Cancer
Source: Cancers (Basel). 2022 May 13;14(10):2399. doi: 10.3390/cancers14102399 (PMC9139200; doi:10.3390/cancers14102399)
Supplement: Supplementary file 1 [file cancers-14-02399-s001.zip › Table S1.zip!/Table S1.pdf]

Table S1. Univariate and multivariate Cox regression proportional hazards model to analyze the correlation between the expression levels of 206 candidate genes and overall survival of patients with cervical cancers.

No.	Gene	Univariate Cox Regression			Multivariate Cox Regression		
		HR	CI95	P Value	HR	CI95	P Value
1	ALOX12B	0.92	0.86-0.99	0.02292	0.84	0.75-0.94	0.00256
2	FAR2	1.27	1.05-1.54	0.01228	1.43	1.11-1.83	0.00501
3	F5	0.79	0.69-0.9	0.00036	0.78	0.65-0.93	0.00599
4	CA9	1.14	1.03-1.26	0.00984	1.16	1.01-1.32	0.03641
5	TDO2	1.17	1.03-1.32	0.01178	1.2	1.01-1.42	0.03654
6	CYP2A13	0.91	0.84-0.98	0.01258	0.9	0.81-1	0.05671
7	HHEX	0.64	0.51-0.8	8.00E-05	0.76	0.56-1.01	0.05952
8	ACP1	1.57	1.02-2.43	0.0422	1.82	0.97-3.42	0.06378
9	HMGCS1	1.24	1.02-1.52	0.0325	1.39	0.97-1.99	0.07568
10	PLA2G3	0.91	0.85-0.97	0.00258	0.92	0.84-1.01	0.07894
11	LDHC	0.94	0.88-1	0.04718	0.92	0.84-1.01	0.08667
12	CA12	1.16	1.03-1.31	0.01785	1.13	0.97-1.33	0.12537
13	SCNN1B	0.9	0.84-0.98	0.00946	1.1	0.96-1.26	0.1817
14	UPP1	1.23	1.01-1.5	0.04216	1.24	0.9-1.71	0.19217
15	DNMT1	0.59	0.4-0.89	0.01049	0.64	0.31-1.33	0.22963
16	GAD1	0.86	0.77-0.95	0.00281	0.93	0.81-1.08	0.35458
17	MMP1	1.13	1.05-1.23	0.00203	1.06	0.93-1.21	0.38451
18	TNFRSF1B	0.8	0.65-0.98	0.0347	1.19	0.79-1.78	0.41458
19	PFKFB4	1.35	1.09-1.66	0.005	0.85	0.58-1.25	0.41989
20	GGT6	0.89	0.79-1	0.04761	1.08	0.89-1.31	0.42498
21	P4HA1	1.58	1.26-1.98	6.00E-05	1.17	0.77-1.79	0.45728
22	POLE2	0.69	0.5-0.95	0.02453	1.2	0.72-2	0.47872
23	PRKCB	0.79	0.68-0.92	0.00328	0.9	0.68-1.21	0.49509
24	SERPINE1	1.2	1.06-1.36	0.00379	0.92	0.72-1.17	0.50341
25	CDA	1.15	1.04-1.27	0.00803	1.06	0.89-1.26	0.52974
26	LIPG	1.21	1.06-1.37	0.00356	0.94	0.79-1.13	0.54082
27	PHKG2	0.56	0.32-0.97	0.04028	0.79	0.37-1.71	0.55584
28	ANGPTL4	1.18	1.03-1.34	0.01666	1.04	0.86-1.27	0.67697
29	GFPT2	1.14	1-1.3	0.04375	1.03	0.84-1.27	0.74699
30	PLAUR	1.36	1.07-1.74	0.01271	0.95	0.66-1.36	0.76936
31	POLE	0.56	0.38-0.83	0.00377	0.96	0.52-1.77	0.89694
32	PLAU	1.24	1.05-1.47	0.0117	1	0.77-1.31	0.98096
33	ACAA1	1.42	0.97-2.09	0.07188	/	/	/
34	ACOT8	0.96	0.66-1.41	0.84287	/	/	/
35	ACOX2	1.12	0.97-1.29	0.11504	/	/	/
36	ACOX3	0.84	0.6-1.19	0.32746	/	/	/
37	ACYP1	0.74	0.53-1.04	0.0798	/	/	/
38	ADRB1	0.95	0.86-1.05	0.35898	/	/	/
39	AGMAT	1.08	0.88-1.34	0.44844	/	/	/

40	AKR1B1	0.95	0.79-1.13	0.54183	/	/	/
41	AKR1B10	0.96	0.9-1.03	0.22184	/	/	/
42	AKR1C2	0.97	0.91-1.03	0.28059	/	/	/
43	AKR1C3	0.96	0.87-1.05	0.37998	/	/	/
44	ALDH2	0.89	0.74-1.08	0.25558	/	/	/
45	ALDH3B2	1.03	0.91-1.16	0.62915	/	/	/
46	ALDH4A1	0.84	0.68-1.04	0.10765	/	/	/
47	ALOX12	0.97	0.88-1.07	0.56117	/	/	/
48	ALOX15B	0.97	0.89-1.06	0.53359	/	/	/
49	ANPEP	1.06	0.95-1.18	0.3248	/	/	/
50	AQP1	0.84	0.67-1.06	0.14178	/	/	/
51	ARG1	1.01	0.94-1.09	0.70437	/	/	/
52	ARG2	0.92	0.78-1.08	0.29666	/	/	/
53	ASNS	1.01	0.8-1.27	0.92036	/	/	/
54	ATP2B1	1.13	0.91-1.39	0.27168	/	/	/
55	BDKRB2	1.16	0.96-1.4	0.11698	/	/	/
56	BLVRB	0.9	0.67-1.2	0.47639	/	/	/
57	C1QB	0.89	0.79-1.02	0.08679	/	/	/
58	C1QC	0.91	0.79-1.04	0.15592	/	/	/
59	C1S	1.1	0.94-1.29	0.24134	/	/	/
60	C3AR1	0.94	0.8-1.1	0.4508	/	/	/
61	C4BPA	0.99	0.94-1.04	0.65202	/	/	/
62	C5	0.86	0.72-1.02	0.0811	/	/	/
63	CBR1	0.96	0.76-1.21	0.74743	/	/	/
64	CBR3	0.87	0.73-1.03	0.10213	/	/	/
65	CBS	0.99	0.93-1.04	0.64964	/	/	/
66	CD38	0.89	0.79-1.01	0.07389	/	/	/
67	CEL	0.93	0.84-1.02	0.11255	/	/	/
68	CES2	0.97	0.78-1.2	0.77519	/	/	/
69	CFD	0.94	0.8-1.1	0.44278	/	/	/
70	CHP1	1.1	0.75-1.62	0.62423	/	/	/
71	CHUK	1.05	0.73-1.5	0.81048	/	/	/
72	CMPK2	0.92	0.79-1.07	0.29804	/	/	/
73	COMT	1.06	0.71-1.56	0.78151	/	/	/
74	CROT	1.05	0.83-1.31	0.69832	/	/	/
75	CTH	1.12	0.95-1.33	0.1646	/	/	/
76	CTPS1	1.41	0.98-2.01	0.06173	/	/	/
77	CYP2C18	0.94	0.87-1.01	0.10557	/	/	/
78	CYP2C9	0.98	0.93-1.04	0.54008	/	/	/
79	CYP2J2	1.13	0.98-1.3	0.09343	/	/	/
80	CYP3A4	0.97	0.9-1.06	0.55284	/	/	/
81	CYP3A5	1.06	0.97-1.17	0.21258	/	/	/
82	CYP4F2	1.01	0.94-1.08	0.827	/	/	/
83	CYP4F3	0.92	0.85-1	0.0633	/	/	/

84	DCK	1.02	0.8-1.29	0.89401	/	/	/
85	DDO	0.99	0.87-1.13	0.87553	/	/	/
86	DECR2	0.96	0.72-1.29	0.79106	/	/	/
87	DGAT2	1.09	0.91-1.32	0.34497	/	/	/
88	DHRS9	0.94	0.84-1.04	0.22615	/	/	/
89	DUT	1.02	0.71-1.46	0.92828	/	/	/
90	ENO2	1.15	0.95-1.4	0.149	/	/	/
91	ENO3	0.94	0.75-1.19	0.62733	/	/	/
92	ENPP3	0.97	0.89-1.06	0.46776	/	/	/
93	ENTPD1	0.95	0.73-1.25	0.73194	/	/	/
94	ENTPD3	1.1	0.96-1.26	0.17445	/	/	/
95	EPHX2	0.89	0.78-1.02	0.0961	/	/	/
96	F12	0.98	0.8-1.19	0.82835	/	/	/
97	F2R	1.12	0.93-1.34	0.24932	/	/	/
98	F3	1.05	0.93-1.2	0.41234	/	/	/
99	FMO1	0.97	0.87-1.07	0.52428	/	/	/
100	FMO2	0.97	0.9-1.05	0.46164	/	/	/
101	GALE	1.21	0.91-1.59	0.18889	/	/	/
102	GCAT	0.8	0.64-1.01	0.0574	/	/	/
103	GCLM	1.04	0.85-1.25	0.72407	/	/	/
104	GLS	1.19	0.94-1.51	0.15571	/	/	/
105	GLUD2	1.05	0.91-1.22	0.49789	/	/	/
106	GMPS	0.93	0.66-1.32	0.69386	/	/	/
107	GNA15	1.04	0.87-1.24	0.67532	/	/	/
108	GNAL	0.92	0.79-1.07	0.27158	/	/	/
109	GPAT2	0.96	0.89-1.03	0.2307	/	/	/
110	GPX3	1.05	0.92-1.19	0.46902	/	/	/
111	GSTA4	0.94	0.83-1.07	0.37221	/	/	/
112	GSTO2	1.06	0.89-1.26	0.52154	/	/	/
113	GSTP1	0.95	0.7-1.28	0.73668	/	/	/
114	GUSB	1.19	0.85-1.66	0.30244	/	/	/
115	GYS2	1.05	0.95-1.16	0.37472	/	/	/
116	HAL	0.99	0.86-1.13	0.85387	/	/	/
117	HEXA	1.41	0.9-2.18	0.13075	/	/	/
118	HPRT1	1.03	0.73-1.47	0.85506	/	/	/
119	HSD11B1	1.05	0.91-1.22	0.47235	/	/	/
120	HTR4	0.94	0.85-1.04	0.25744	/	/	/
121	INPP4A	0.98	0.75-1.28	0.879	/	/	/
122	INPP5K	0.75	0.46-1.23	0.25547	/	/	/
123	ITPR2	1.12	0.91-1.38	0.28082	/	/	/
124	ITPR3	1.29	0.98-1.7	0.06466	/	/	/
125	LAP3	0.95	0.74-1.21	0.6599	/	/	/
126	MAOA	0.96	0.85-1.08	0.45416	/	/	/
127	MAOB	0.97	0.86-1.09	0.56002	/	/	/

128	MAPK10	0.97	0.86-1.1	0.62638	/	/	/
129	MASP1	0.92	0.83-1.03	0.13878	/	/	/
130	MBOAT2	1.14	0.95-1.36	0.17337	/	/	/
131	MGLL	1.17	0.98-1.4	0.0774	/	/	/
132	MINPP1	1.27	0.96-1.69	0.09839	/	/	/
133	MPST	1.07	0.81-1.41	0.64038	/	/	/
134	MTHFD1	0.74	0.46-1.19	0.2117	/	/	/
135	MTHFD2	1.17	0.83-1.63	0.37575	/	/	/
136	MTR	1.18	0.89-1.56	0.24574	/	/	/
137	MVK	0.84	0.58-1.21	0.34662	/	/	/
138	NANP	1.02	0.76-1.37	0.88082	/	/	/
139	NEDD4L	1.02	0.78-1.33	0.91328	/	/	/
140	NEU1	1.19	0.89-1.59	0.24049	/	/	/
141	NNMT	1.12	0.99-1.26	0.07773	/	/	/
142	NNT	1.05	0.82-1.34	0.69072	/	/	/
143	NT5C2	1.01	0.77-1.34	0.9293	/	/	/
144	OPLAH	1.18	0.92-1.52	0.18959	/	/	/
145	P2RX5	0.96	0.85-1.09	0.55214	/	/	/
146	PAX6	0.96	0.87-1.05	0.35889	/	/	/
147	PCYT1A	1.04	0.79-1.37	0.77055	/	/	/
148	PCYT2	0.87	0.59-1.27	0.45994	/	/	/
149	PDE10A	0.98	0.88-1.1	0.74756	/	/	/
150	PDE1A	1.01	0.85-1.19	0.95327	/	/	/
151	PDE4B	1.07	0.9-1.26	0.44288	/	/	/
152	PDE7A	0.99	0.76-1.3	0.95636	/	/	/
153	PEX11G	0.9	0.69-1.16	0.41228	/	/	/
154	PGM2L1	1.04	0.89-1.22	0.62537	/	/	/
155	PHGDH	1.01	0.88-1.16	0.85695	/	/	/
156	PIK3C2B	1.04	0.84-1.29	0.74393	/	/	/
157	PIK3CA	1.13	0.89-1.43	0.3277	/	/	/
158	PIK3CG	0.94	0.8-1.11	0.48199	/	/	/
159	PIK3R1	1.01	0.87-1.17	0.90451	/	/	/
160	PIK3R2	1.02	0.75-1.4	0.89185	/	/	/
161	PIK3R3	0.85	0.69-1.05	0.12931	/	/	/
162	PLA2G4A	1.09	0.96-1.23	0.18131	/	/	/
163	PLA2G6	0.87	0.71-1.07	0.19585	/	/	/
164	PLA2G7	0.87	0.75-1.01	0.07579	/	/	/
165	PLCB1	1.05	0.92-1.2	0.43576	/	/	/
166	PNPT1	1.41	1-1.99	0.05001	/	/	/
167	POLA1	0.82	0.62-1.07	0.13905	/	/	/
168	POLD3	0.94	0.65-1.34	0.72307	/	/	/
169	POLR3K	0.76	0.46-1.24	0.27183	/	/	/
170	PPP3R1	1.14	0.77-1.67	0.51717	/	/	/
171	PRIM1	0.87	0.63-1.21	0.41456	/	/	/

172	PRIM2	0.85	0.59-1.22	0.37639	/	/	/
173	PRKCA	1.17	0.98-1.38	0.07571	/	/	/
174	PRPS1	0.94	0.68-1.29	0.68719	/	/	/
175	PRPS2	0.81	0.64-1.03	0.08953	/	/	/
176	PSAT1	0.98	0.8-1.18	0.80499	/	/	/
177	PTDSS1	1.22	0.89-1.69	0.21972	/	/	/
178	QPRT	1.05	0.94-1.16	0.40379	/	/	/
179	RDH12	0.97	0.88-1.06	0.48391	/	/	/
180	RDH16	1.1	0.97-1.23	0.12464	/	/	/
181	RRM1	0.91	0.65-1.27	0.56143	/	/	/
182	RRM2	1.28	0.97-1.68	0.07735	/	/	/
183	RYR1	0.95	0.87-1.03	0.19283	/	/	/
184	SCD5	0.94	0.8-1.09	0.40533	/	/	/
185	SCNN1A	0.96	0.85-1.08	0.50517	/	/	/
186	SCNN1G	0.94	0.88-1.01	0.08534	/	/	/
187	SGPP2	1	0.88-1.15	0.96693	/	/	/
188	SLC25A17	0.89	0.62-1.27	0.52043	/	/	/
189	SLC27A1	0.93	0.72-1.2	0.58174	/	/	/
190	SLC27A4	0.93	0.67-1.31	0.68587	/	/	/
191	SLC27A6	1.02	0.94-1.09	0.68134	/	/	/
192	SMPD2	1.08	0.76-1.52	0.66734	/	/	/
193	SMS	0.99	0.73-1.32	0.92435	/	/	/
194	SOCS3	1.09	0.9-1.33	0.37469	/	/	/
195	SPHK1	0.97	0.78-1.2	0.76732	/	/	/
196	SULT2B1	0.95	0.84-1.07	0.40669	/	/	/
197	SUOX	0.99	0.73-1.32	0.92199	/	/	/
198	SYNJ1	0.97	0.74-1.28	0.83134	/	/	/
199	TK1	0.82	0.6-1.12	0.21395	/	/	/
200	TXNDC12	1.57	0.9-2.74	0.11196	/	/	/
201	TXNRD1	1.01	0.83-1.23	0.94334	/	/	/
202	TYMS	0.82	0.62-1.1	0.18753	/	/	/
203	UGT2B17	0.95	0.88-1.02	0.14906	/	/	/
204	UGT2B28	0.91	0.82-1.01	0.08189	/	/	/
205	UGT8	1	0.94-1.07	0.93154	/	/	/
206	UMPS	0.91	0.64-1.29	0.60355	/	/	/
